# *Moringa oleifera* Improves MAFLD by Inducing Epigenetic Modifications

**DOI:** 10.3390/nu14204225

**Published:** 2022-10-11

**Authors:** C. Alejandra Monraz-Méndez, Rebeca Escutia-Gutiérrez, Jonathan Samael Rodriguez-Sanabria, Marina Galicia-Moreno, Hugo Christian Monroy-Ramírez, Laura Sánchez-Orozco, Jesus García-Bañuelos, Ricardo De la Rosa-Bibiano, Arturo Santos, Juan Armendáriz-Borunda, Ana Sandoval-Rodríguez

**Affiliations:** 1 Institute for Molecular Biology in Medicine and Gene Therapy, Department of Molecular Biology and Genomics, Health Sciences University Center, University of Guadalajara, Guadalajara 44340, Jalisco, Mexico; 2 Tecnologico de Monterrey, Escuela de Medicina, Monterrey 64849, Nuevo Leon, Mexico

**Keywords:** MAFLD, epigenetics, Moringa, miRNAs, liver, microarrays

## Abstract

Background and aims. Metabolic Associated Fatty Liver Disease (MAFLD) encompasses a spectrum of diseases from simple steatosis to nonalcoholic steatohepatitis (NASH). Here, we investigated the hepatoprotective role of *Moringa oleifera* aqueous extract on hepatic miRNAs, genes and protein expression, as well as histological and biochemical parameters in an experimental model of NASH. Methods. Male C57BL/6J mice were fed with a high fat diet (HFD, 60% lipids, 42 gr/L sugar in water) for 16 weeks. Moringa extract was administered via gavage during the final 8 weeks. Insulin Tolerance Test (ITT) and HOMA-IR were calculated. Serum levels of insulin, resistin, leptin and PAI-1 and hepatic expression of *miR-21a-5p*, *miR-103-3p*, *miR-122-5p*, *miR-34a-5p* and SIRT1, AMPKα and SREBP1c protein were evaluated. Alpha-SMA immunohistochemistry and hematoxylin-eosin, Masson’s trichrome and sirius red staining were made. Hepatic transcriptome was analyzed using microarrays. Results. Animals treated with Moringa extract improved ITT and decreased SREBP1c hepatic protein, while SIRT1 increased. Hepatic expression of *miR-21a-5p*, *miR-103-3p* and *miR-122-5p*, *miR34a-5p* was downregulated. Hepatic histologic analysis showed in Moringa group (HF + MO) a significant decrease in inflammatory nodules, macro steatosis, fibrosis, collagen and αSMA reactivity. Analysis of hepatic transcriptome showed down expression of mRNAs implicated in DNA response to damage, endoplasmic reticulum stress, lipid biosynthesis and insulin resistance. Moringa reduced insulin resistance, de novo lipogenesis, hepatic inflammation and ER stress. Conclusions. Moringa prevented progression of liver damage in a model of NASH and improved biochemical, histological and hepatic expression of genes and miRNAs implicated in MAFLD/NASH development.

## 1. Introduction

Metabolic Associated Fatty Liver Disease (MAFLD) is characterized by hepatic steatosis accompanied by one of three features: overweight or obesity, T2DM (Type 2 Diabetes Mellitus), or lean or normal weight with evidence of metabolic dysregulation [1].

MAFLD, as with the previous term NAFLD, represents the hepatic manifestation of a multisystemic disorder, with prevalence of 20–35% in western countries [2]. The mechanisms underlying the development and progression of NAFLD are displayed in a multiple hit model that involves IR, nutritional factors overload, gut microbiota dysfunction, endoplasmic reticulum (ER) stress, inflammatory liver environment and genetic and epigenetic factors [3]. Changes in diet and increase in physical activity are the first line of current treatment of hepatic steatosis [4]. The scarce number of pharmacological therapeutic agents for MALFD has led to many bioactive molecules, such as phenolic compounds present in plants, being tested as MALFD therapies.

One of these studied plants is Moringa oleifera. Scientists have reported the medicinal effects of Moringa leaf extracts in different animal and cell models due to their anti-inflammatory, lipid-lowering and antioxidant effects [5,6,7,8]. The mechanisms of action of Moringa extracts in steatohepatitis models so far described, mainly comprise a reduction in lipogenesis and/or increase in the oxidation of fatty acids, in addition to its antioxidant effects [5,9,10].

More than 1800 miRNAs have been identified in humans with approximately 45,000 target genes. It is considered that the expression of more than 60% of a human cell’s total proteins are regulated by miRNAs [11]. Hepatic miRNAs regulate various metabolic pathways, such as glucose and lipid metabolism, inflammation, apoptosis and necrosis of hepatocytes, and fibrosis [12]. Alterations in liver metabolism implicated in MALFD/NASH are attributed to dysregulations in the expression of miRNAs. miR-122 is the most abundant and studied hepatic miRNA representing 70% of the total hepatic miRNAs and interacts with numerous target genes involved in lipid and cholesterol metabolism [11,13]. Latorre et al. showed that miR-16, miR-30b and miR-30c are involved in lipid homeostasis and down-regulated in hepatocytes with fat accumulation. miR-21 regulates triglycerides, free cholesterol and total cholesterol levels. In addition, the elevation of miR-21 induces fatty acid uptake and lipid accumulation [14,15]. Xu et al. demonstrated that miR-34a inhibits hepatic VLDL secretion by promoting steatosis in mice fed with HFD. miR-103 regulates insulin sensitivity and glucose homeostasis and is highly expressed in the liver of patients with NAFLD [16]. An overexpression of miR-103 results in IR.

Additionally, it has been reported that different polyphenols, such as resveratrol and pro-anthocyanidins, modify the expression of miR-122-5p, miR-103-3p and miR-21a-5p [17,18,19]. Gracia et al. demonstrated that overexpression of miR-103, miR-107 and mir-122 in AML12 hepatocytes increases the expression of sterol regulatory element-binding protein 1 (SREBP1c). On the other hand, in HFD rats treated with synthetic resveratrol, the expression of these miRNAs decreases in parallel with a reduction of SREBP1c protein. Administration of pro-anthocyanidin extract from grape seed reverses the increase in miR-122 in obese rats [18]. Plant-derived polyphenols extract (*Hibiscus sabdariffa*) regulates expression of miRNAs miR103/107 and miR-122 and prevents diet-induced fatty liver disease in hyperlipidemic mice [17]. To the best of our knowledge, this is the first communication where the effect of Moringa extracts is represented in epigenetic modifications by modulating microRNA (miRNA) expression in MALFD/NASH models.

Our major thrust was to determine the effect of Moringa extract in epigenetic modifications, specifically, the expression of miRNAs implicated in MAFLD/NASH development and its outcome on hepatic transcriptome in order to elucidate molecular mechanisms involved in the pathogenesis of MALFD/NASH.

## 2. Materials and Methods

Preparation and characterization of the aqueous extract of *Moringa oleifera*.

The powdered leaves of *Moringa oleifera* were obtained from the South Coast of Jalisco and were validated by the National Institute of Forestry, Agricultural and Livestock Research (INIFAP). Powder was mixed with sterile water, at a concentration: 62.5 mg/mL. The mix was left for 65 h at 4 °C in the dark to obtain the aqueous extract. Two centrifugations were carried out at 13,000× *g* for 10 min, and supernatant was collected. Extract was stored at −80 °C until use [6]. Extract characterization was made using 2,2-Difenil-1-Picrilhidrazilo (DPPH) and Diammonium 2,2′-azino-bis(3-ethylbenzothiazoline-6-sulfonate (ABTS) spectrophotometric assays [20,21]. Briefly, DPPH stock (300 μM) reacted with a standard curve of Trolox (10–1000 μM) and samples. After 30 min’s repose, absorbance was measured at 510 nm. For ABTS measurement, a ABTS 7 mM stock solution and K_2_S_2_O_2_ 2.4 mM were incubated overnight with shaking. Absorbance of the solution was adjusted at 0.70 ± 0.01 at 734 nm. ABTS was tested in a standard curve of Trolox (10–1000 μM) and samples. After 6 min’s repose, standard curve and samples were measured at 750 nm. DPPH and ABTS were expressed as μMET.

### 2.1. Experimental Design

Fifteen male mice of the C57BL/6J strain aged 4 weeks weighing 20–25 g were acquired from Cinvestav animal facility (Mexico City, Mexico). Upon arrival, mice received care according to the guidelines of the Animal House of the University Center of Health Sciences of the University of Guadalajara and the Official Mexican Standard NOM-062-ZOO-1999. Mice were kept in polycarbonate boxes in a room at 28 + 1 ° C and an alternating 12-h light/dark cycle, with standard diet on demand and water ad libitum. After a week of acclimatization, mice were separated into two groups at random. Sample size was calculated to detect a difference of 10% between the means of two independent samples, resulting in groups of 5 animals, having a statistical power of 80% and a significance of 95%. The groups were as follows: a group of mice fed a standard diet (ND) (n = 5) (Envigo T.2018S.15, 3.1 Kcal/g, 18% lipids, 58% carbohydrates, 24% proteins) and a group of mice fed a high fat diet (HF) (n = 10) (Envigo TD.06414, 5.1 Kcal/g, 60.3% lipids, 21.4% carbohydrates, 18.3% proteins), in addition to high-sugar drinking water (2.31% g/v fructose and 1.89% g/v sucrose) on free demand for 16 weeks to induce NASH [22,23]. At week 9, the HF group mice was divided into 2 sub-groups (n = 5): HF group without treatment (HF) and HF group administered daily with 150 uL of the Moringa leaves aqueous extract (HF + MO) (62.5 mg/mL). Extract was administered using an orogastric cannula at the same hour of the day, daily for 8 weeks until the end of the study. Final concentration of the extract in mouse was calculated to be 290 mg/Kg. Moringa dose was calculated according to a previous pilot study. Animals were euthanized after 4 h of fasting using anesthesia. The liver and epididymal adipose tissue were collected and weighed. For histological analysis, the three main hepatic lobes and a part of the epididymal fat were fixed in 4% paraformaldehyde/0.1 M PBS, pH 7.4. The rest of the liver and epididymal adipose tissue samples were placed in Eppendorf tubes, flash frozen in nitrogen and stored at −80 °C until analysis. This protocol was approved by the CUCS Ethics, Research and Biosafety Committees: CINV/008/20.

### 2.2. Energy Consumption, Weight, Fasting Glucose, Insulin Sensitivity and Insulin Resistance

Food and drink consumption were systematically measured every other day. Food and drink intake were estimated as follows: 

total food intake per box (gr)/number of mice per box = gr food/mouse/day

7 days of the week

total drink intake per box (mL)/number of mice per box = mL drink/mouse/day

7 days of the week.

The caloric intake was analyzed in two phases, before Moringa administration and during Moringa administration, to assess whether Moringa reduced caloric intake. During the study, weight was measured and fasting glucose concentration was quantified weekly. Blood glucose levels were measured using tail vein puncture with a glucometer (LifeScan Inc. Milpitas, CA, USA) after 4 h of fasting. At the end of the study, 48 h before sacrifice; mice were fasted for 4 h and underwent the insulin tolerance test (ITT) using short-duration recombinant human insulin at a standardized dose of 0.025 U/mouse. Glucose levels were measured at 0, 30, 60 and 90 min after insulin administration. Subsequently, the area under the curve was calculated. Homeostatic Model to evaluate Insulin Resistance (HOMA-IR) was calculated as follows:

HOMA-IR = (fasting glucose (mg/dL) × fasting insulin (uIU/mL))/405.

### 2.3. Biomarkers in Serum

Blood was collected from the ocular vein and centrifuged at 1500× *g*/10′ at 4 °C to obtain serum. Serum aliquots were stored at −80 °C for further analysis. The serum levels of AST, ALT, total cholesterol and VLDL were quantified using VITROS DT60II dry chemistry analyzer (Johnson & Johnson, Nuevo Brunswick, NJ, USA). Insulin, leptin, resistin and PAI-1 were quantified using the multiplex immunoassay kit (Bio-Plex Pro™ Diabetes Assay, BioRad, Hercules, CA, USA) according to manufacturer’s instructions.

### 2.4. Lipid Peroxidation Assessment

The determination of Malondialdehyde (MDA) levels in biological samples is a convenient, sensitive, and widely used method to quantitatively estimate the extent of lipid peroxidation [24]. In our study, MDA was assessed in liver homogenates using the thio-barbituric acid (TBA) method [25]. Briefly, 300 uL of the liver homogenate are mixed with 700 uL of a Tris-HCl 150 mM solution and 2 mL of 0.375% TBA dissolved in 15% trichloroacetic acid (TCA). The mixture is incubated at 95–100 ° C for 45 min in a boiling water bath. At the end of the incubation, the samples are centrifuged at 4000 rpm for 15 min. The MDA-TBA_2_ formed is measured at 532 nm. A correlation was made with the total protein, which was measured by the Bradford method using albumin as a standard [26].

### 2.5. Histological Analysis of Liver

Morphological evaluation and tissue staining were carried out in paraffin-embedded liver sections. Hematoxylin-Eosin (H&E), Masson and Sirius red staining were performed to evaluate inflammation, fibrosis, total steatosis. Twenty (20X) microscopic photographs were analyzed for these purposes. All images were taken with an Olympus BX51 microscope (Center Valley, PA, USA) and analyzed using a computer-assisted image analyzer (Image-ProPlus 6.0, Rockville, MD, USA).

### 2.6. Immunohistochemistry

Liver stellate cell activation was assessed with IHC for alphaSMA (Ab19245, Cell Signaling. Danvers, MA, USA). After incubation and washing, the attached primary antibody did bind to the labeled secondary antibody following the manufacturer’s protocol (PK-8800 VECTASTAIN® Universal Quick HRP Kit, Peroxidase (Vector, Newark, CA, USA). The final reaction was carried out by immersing tissue sections in a 3,3-diaminobenzidine solution (Code K3468 Liquid DAB+, 2-component system, Immunohistochemistry Visualization (Agilent-Dako. Santa Clara, CA, USA) and contrasted staining using hematoxylin.

### 2.7. microRNAs Expression

Total miRNAs were extracted using the miRVana microRNA isolation kit (AM1561, mirVana™ miRNA Isolation Kit; Thermo Fisher Scientific, Waltham, MA, USA) according to the manufacturer’s instructions. miRNAs reverse transcription was carried out using TaqMan Advanced miRNA cDNA Synthesis Kit (A28007, Thermo Fisher Scientific, Waltham, MA, USA). Taqman probes specific for the analyzed miRNAs (Supplemental Table S1) were used (TaqMan Advanced miRNA Assay, Thermo Fisher Scientific, Waltham, MA, USA). Briefly, 2 µg of total miRNA/sample were used. Polyadenylation reaction conditions were incubation for 45 min at 37 °C, 10 min at 65 °C; followed for the adapter ligation reaction: 60 min at 16 °C and retro-transcription at 42 °C for 15 min, incubation at 85 °C for 5 min. Amplification reaction (miR-Amp) was carried out for 15 cycles using 95 °C/3 s for denaturalization and 60 °C/30 s in the annealing. qRT-PCR using Taqman Fast Advanced Master Mix (cat. 4444557) was performed as follows: enzyme activation (95 °C for 20 s) followed by 60 cycles of denaturalization (95 °C/1 s) and annealing (60 °C/20 s) in a Lightcycler 96 equipment (Roche, Rotkreuz, Switzerland). mmu-miR-16-5p was used for normalization of samples using 2ΔCt analysis [27].

### 2.8. Analysis of Gene Expression by Real-Time PCR

RNA extraction was performed in liver homogenate from the 3 main lobes according to the modified method of Chomczynski and Sacchi [28]. RNA was quantified using NanoDrop equipment at 240 nm. Reverse transcription using 2 µg of total RNA was performed with 240 ng Oligo dT, 0.5 mM dNTPs mix, 10 mM DTT, 2U of RNAse inhibitor and 200U M-MLV, applying incubation for 10 min at 25 °C, 50 min at 37 °C, 15 min at 70 °C and 5 min on ice. Samples were stored at −80 °C until use. qPCR was performed under the following conditions: 50 ° C for 2 min, 95 ° C for 5 min, and 40 cycles at 95 °C for 30 s and at 60 °C for 40 s. The total volume of the reaction was 10 µL containing 2 µL of cDNA, 1X Universal PCR Master Mix (cat. #4461882) and specific 1X TaqMan probes (Supplemental Table S2) (Thermo Fisher Scientific, Waltham, MA, USA). GADPH was used as housekeeping gene. Analysis was performed using the 2Δct method [27].

### 2.9. Western Blot Analysis

Total protein extraction was performed in liver samples using RIPA followed by Bradford quantification. Aliquots (20 ug) of extracted total protein were loaded onto a 10% SDS-PAGE gel under reducing conditions and were then transferred onto polyvinylidene difluoride (PVDF) membrane (Bio-Rad Laboratories, Inc., Hercules, CA, USA), which were blocked with 10% milk powder containing 0.1% Tween 20 at 4 °C, overnight. Subsequently, membranes were immunoblotted with specific antibodies (1:1000) overnight at 4 °C (Supplemental Table S3). Afterward, membranes were incubated with a peroxidase-conjugated secondary antibody (1:16,000) for 1 h at room temperature. Band analysis was carried out using Image Lab 5.0 software (BioRad, Hercules, CA, USA). β-Tubulin was used as loading control (1:5000).

### 2.10. Transcriptome Analysis

Double-channel microarrays for the complete *M. musculus* genome were realized. Comparison was carried out as follows: HF vs. ND and HF + MO vs HF. genArise analysis tool was used for image analysis. Bioinformatics analysis included DAVID Bioinformatics Resources 6.8 platform (https://david.ncifcrf.gov/, accessed on 20 May 2020) and Gene Ontology Biological Process database.

### 2.11. Statistical Analysis

Data is expressed as mean ± standard error. Quantitative variables were analyzed with one-way ANOVA and post-hoc with the Tukey test for parametric data, Kruskal-Wallis and Mann-Whitney U for non-parametric data. GraphPad Prism software version 10 was used (San Diego, CA, USA). A *p* < 0.05 was considered statistically significant.

## 3. Results

### 3.1. Moringa Extract Showed Antioxidant Capacity

The antioxidant capacity of Moringa aqueous extract was characterized. DPHH value was 10,081.4 ± 0.3; while ABTS values were 22,960.4 ± 0.3 indicating a natural source of polyphenols with antioxidant capacity.

### 3.2. Moringa Diminished Animal, Liver and Epididymal Fat Weight 

**Figure 1:**
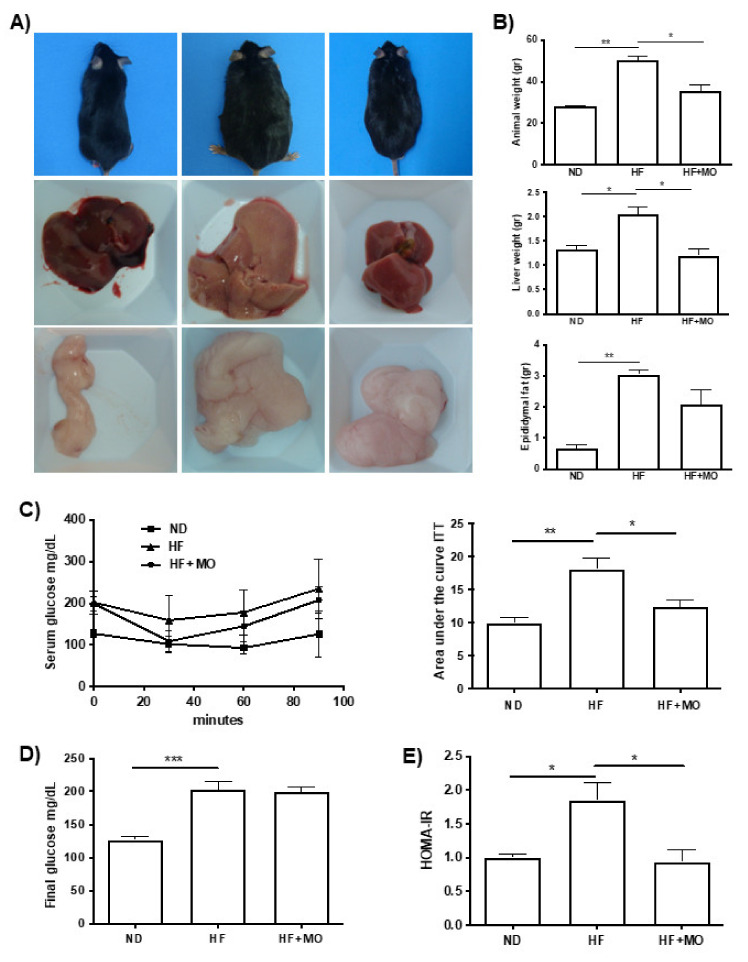
Moringa diminished animal, liver and epididymal fat weight and maintained insulin sensitivity in animals feed with HF diet. (**A**) Representative photographs of mice, liver and epididymal fat. (**B**) Graphs of animal, liver and epididymal fat weight showed a reduction in MO group (*p* < 0.05). (**C**) ITT demonstrated a significant improvement in insulin sensitivity in MO group compared to HF animals (*p* < 0.05). (**D**) Serum glucose levels at sacrifice were similar in MO and HF groups. (**E**) HOMA-IR showed insulin resistance in present only in HF animals (*p* < 0.05). Data are expressed as mean ± SE. * *p* < 0.05; ** *p* < 0.01; *** *p* < 0.001.

Macroscopic photographs in Figure 1A showed that liver and epididymal fat pad in MO treated group had significant reduction, as well as animal weight. At the time of sacrifice, HF group diet had a higher animal weight compared to the control group (ND) with a standard diet (50.14 g ± 2.93 g vs. 27.98 g ± 0.47 g; *p* < 0.01). While HF + MO group showed a significantly lower animal weight (50.14 gr ± 2.93 gr vs. 35.52 gr ± 2.98 gr; *p* < 0.05; Figure 1B). Liver weight at the time of sacrifice increased significantly (2.04 gr ± 0.17 gr vs. 1.33 gr ± 0.08 gr; *p* < 0.05) in HF group, while HF + MO group presented a reduction (2.037 gr ± 0.17 gr vs. 1.195 gr ± 0.15 gr; *p* < 0.05; Figure 1B), reaching values comparable to ND group. Epididymal fat pad weight was significantly higher in HF group compared with ND group (3.05 gr ± 0.14 gr vs. 0.67 gr ± 0.12 gr; *p* < 0.01); and group treated with Moringa extract exhibited a tendency to reduce compared to HF animals (Figure 1B).

### 3.3. Moringa Reduced Daily Food Intake

As shown in Table 1, dietary intake was calculated in the three different groups during the study. Energy consumption (Kcal) during the study was significantly higher in HF-fed group compared to ND group. HF and ND groups did not change in any phase of the study. Prior to treatment, HF + MO group showed similar daily energy consumption (Kcal) and daily food consumption (g) than HF animals. However, during MO extract administration, food consumption (g) and daily energy consumption (Kcal) decreased significantly in this group (*p* < 0.05).

### 3.4. Moringa Preserves Insulin Sensitivity in MAFLD/NASH Animals

As shown in Figure 1D, the group that followed a HF diet had significantly higher serum glucose levels during the study compared to the ND group (202 mg/dL ± 12.57 vs. 127 mg/dL ± 4.92; *p* < 0.001). However, no significant differences were found in serum glucose levels between HF and HF + MO group. However, when performing the ITT at the end of the study, HF + MO group showed greater sensitivity to insulin compared to the HF group (18.09 ± 1.69 vs. 12.35 ± 1.12; *p* < 0.05; Figure 1C), reaching similar levels to ND group; despite presenting major basal levels. Improvement in insulin sensitivity was corroborated calculating HOMA-IR, which was significantly reduced in the HF + MO treated group compared to the HF group (*p* < 0.05; Figure 1E). HOMA-IR has been widely used for the estimation of insulin resistance in research. The higher the number, the more resistant to insulin.

### 3.5. Moringa Improves Biochemical Hepatic Test and Adipokines Serum Levels

**Figure 2:**
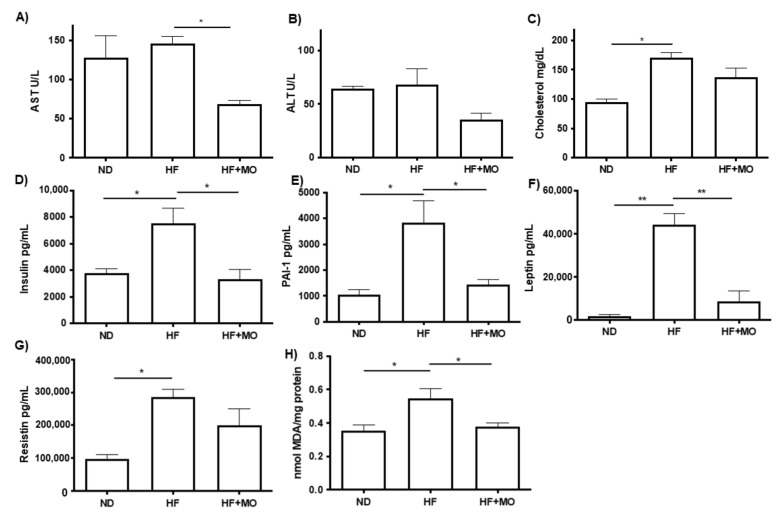
Supplementation of Moringa improves biochemical hepatic test, adipokines serum levels and lipid peroxidation. (**A**,**B**) Hepatic enzymes showed reduction in Mo group, especially AST (*p* < 0.05). (**C**) Cholesterol serum levels did not show statistical difference between HF and Mo groups. Serum adipokines (**D**) Insulin, (**E**) Plasminogen activator inhibitor-1, (**F**) Leptin and (**G**) Resistin decreased in Mo animals (*p* < 0.05 and *p* < 0.01). (**H**) MDA hepatic levels indicated a reduced lipid peroxidation in animals treated with Mo. Data are expressed as mean ± SE. * *p* < 0.05; ** *p* < 0.01.

The group treated with Moringa showed reduction in AST and ALT levels compared to HF group, though significant reduction in AST levels was seen in the HF + MO group against the HF group (68.80 U/L ± 4.26 vs. 146.3 U/L ± 8.84 U/L; *p* < 0.05; Figure 2A). Total cholesterol increased significantly in the HF group compared to the ND group (170.8 mg/dL ± 8.5 mg/dL vs. 95.3 mg/dL ± 5.2; *p* < 0.05; Figure 2C), the HF + MO treated group had a reduction in cholesterol, though not significant compared with HF group.

**Figure 3:**
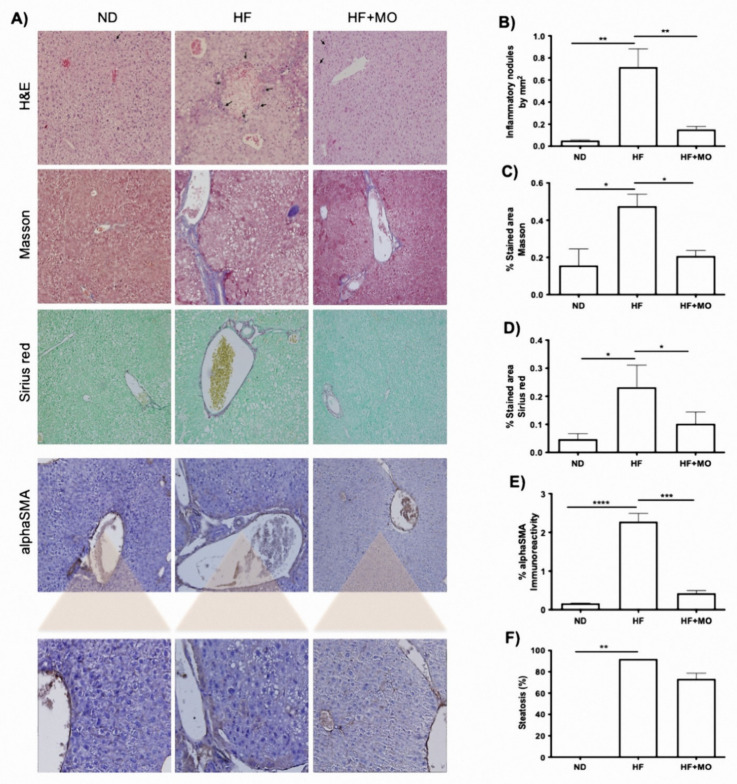
Liver histology showed improved parameters after Mo supplementation. (**A**) Representative microphotographs (40X) of liver tissue stained with H&E, Masson, Sirius Red and after IHQ against αSMA. (**B**) Quantity of inflammatory nodules is reduced in Mo animals compared to HF group (*p* < 0.01). (**C**,**D**) showed a decrease in ECM and collagens after Mo supplementation (*p* < 0.05). (**E**) Positivity to αSMA is increased only in HF animals (*p* < 0.001). (**F**) steatosis tent to be reduced in Mo animals. Data are expressed as mean ± SE. * *p* < 0.05; ** *p* < 0.01; *** *p* < 0.001; **** *p* < 0.0001.

The serum levels of insulin, resistin, leptin and PAI-1 are significantly increased in the HF groups compared to the control group (Figure 3D–F). Noteworthy, insulin (7505 pg/mL ± 943.1 vs. 3335 pg/mL ± 889.2; *p* < 0.05, Figure 2D), PAI-1 (3824 pg/mL ± 867.2 vs. 1425 pg/mL ± 209.3; *p* < 0.05, Figure 2E) and leptin serum levels (44,373 pg/mL ± 5129 vs. 8718 pg/mL ± 4872; *p* < 0.01, Figure 2F) were significantly lower in the HF + MO group compared to the HF group. Serum levels of resistin (Figure 2G) did not show significant changes in the group treated with Moringa, though a tendency to decrease was observed.

### 3.6. Moringa Reduced Lipid Peroxidation

To evaluate oxidative damage in the liver, lipid peroxidation was determined by measuring MDA content in liver samples. As shown in Figure 2H, MDA levels increased significantly in the HF group against ND group (*p* < 0.05), indicating an increase in lipoperoxidation. The group treated with moringa significantly reduced MDA levels (*p* < 0.05), reaching levels comparable to ND group.

### 3.7. Liver Histology Improved after Moringa Administration

As shown in the representative images of the histological analysis (Figure 3A), a significant increase in both inflammatory nodules and periportal and centrilobular fibrosis is observed in the HF group against ND group (*p* < 0.05). Also, there is an increase in macro and micro-vesicular steatosis distributed in all liver zones, and present in more than 80% of the tissue in HF group. This steatosis is not observed in ND livers (*p* < 0.01). On HF + MO animals, there is a decrease in macro-vesicular steatosis, and micro-steatosis persist in liver zones 2 and 3.

Figure 3B shows a significant diminution in the number of inflammatory nodules between the HF + MO group and the HF group (*p* < 0.01). Extracellular matrix quantification performed in Masson’s staining denoted a significant reduction in the HF + MO group compared to the HF group (*p* < 0.05; Figure 3C). Likewise, specific staining for collagens with Sirius red, revealed a significant decrease in HF + MO treated group (*p* < 0.05; Figure 3D). By immunohistochemistry, the presence of αSMA in the liver was analyzed, showing a significant increase in the reactivity of the HF group compared to the ND group (*p* < 0.0001; Figure 3E). In the group treated with Moringa, the activation of Hepatic stellate cells (HSC) to the myofibroblast phenotype significantly decreased (HF + MO vs. HF, *p* < 0.001; Figure 3E).

### 3.8. Moringa Modifies Hepatic miRNAs Expression

**Figure 4:**
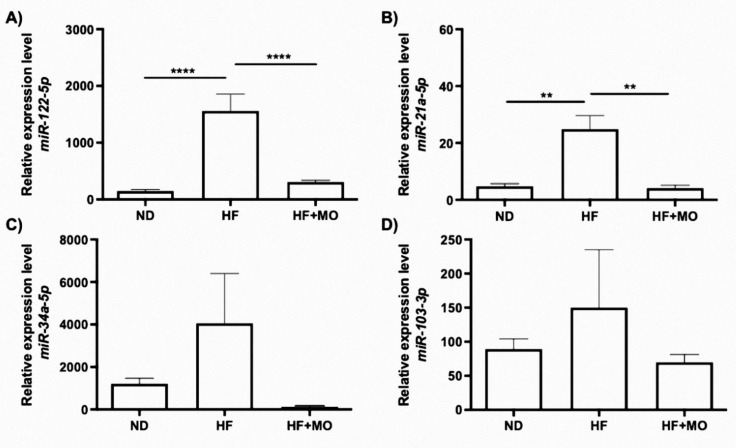
Hepatic levels of miRNAs implicated in MAFLD pathogenesis. Gene expression of (**A**) miR-122-5p (*p* < 0.0001), (**B**) miR-21a-5p (*p* < 0.01), (**C**) *miR-34a-5p* and (**D**) *miR-103a-3p* tend to reduce in Mo animals compared to HF group. Data are expressed as mean ± SE. ** *p* < 0.01; **** *p* < 0.0001.

The levels of *miR-122-5p*, *miR-21a-5p*, *miR-34a-5p* and *miR-103-3p* tend to be higher in the HF group compared to the ND control group (Figure 4A–D). Moringa treatment significantly reduces the levels of *miR-122-5p* and *miR21a-5p* (*p* < 0.05; Figure 4A,B). However, there was no significant reduction in *miR-34a-5p* and *miR-103-3p* levels in the group treated with moringa, but a tendency to decrease expression was observed (Figure 4C,D).

### 3.9. Moringa Extract Reduces Expression of Inflammation-Related Genes

**Figure 5:**
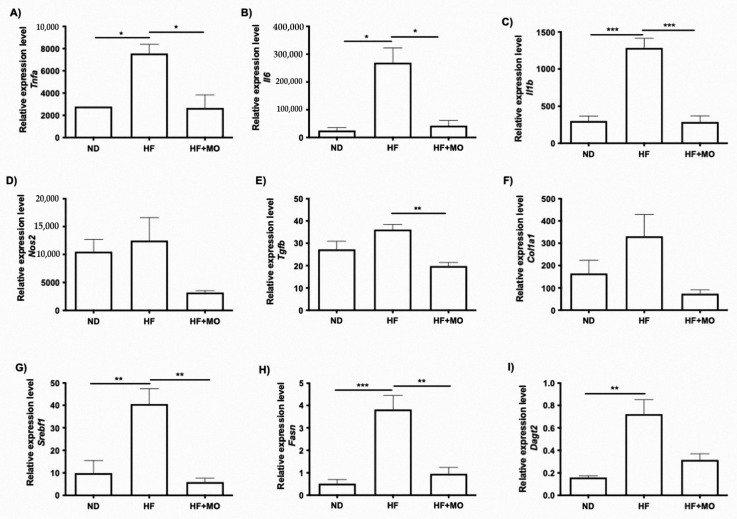
Hepatic mRNA levels in molecules involved in inflammation, fibrosis and lipid metabolism. Proinflammatory gene expression (**A**) Tnfa (*p* < 0.05) (**B**) Il6 (*p* < 0.05) (**C**) Il1b (*p* < 0.001) and (**D**) *Nos2* are reduced after Mo supplementation compared to HF animals (*p* < 0.05 and *p* < 0.01). mRNA levels of the main fibro-genic molecules (**E**) *Tgfb* (*p* < 0.01) and (**F**) *Srebf1* (*p* < 0.01) tent to be increase only in HF animals. Lipogenic genes evaluated: (**G**) *Srebf1*, (**H**) *Fasn* (*p* < 0.01), and (**I**) *Dagt2* showed a reduction in Mo treated animals compared to HF group (*p* < 0.05). Data are expressed as mean ± SE. * *p* < 0.05; ** *p* < 0.01; *** *p* < 0.001.

The expression of genes involved in inflammatory processes such as *Il1b*, *Il6* and *Tnfa*, was analyzed in liver tissue. mRNA levels of *Il1b*, *Il6* and *Tnfa* were higher in HF group compared to ND group (Figure 5A–C). Moringa treatment significantly reduced *Tnfa*, *Il6* (*p* < 0.05; Figure 5A,B) and *Il1b* levels (*p* < 0.001; Figure 5C). *Nos2* expression in the group treated with Moringa did clearly tend to decrease (Figure 5D).

### 3.10. Moringa Extract Reduces Fibrosis-Related Genes Expression

Gene expression in liver fibrosis-related molecules such as *Tgfb* and *Col1a1* was analyzed. *Tgfb* and *Col1a1* mRNA levels tend to be higher in HF group compared to ND group (Figure 5E,F). Moringa treatment significantly reduced *Tgfb* levels (*p* < 0.01; Figure 5E). However, there was no significant reduction in *Col1a1* mRNA levels in the groups treated with Moringa, though a decreased gene expression was observed (Figure 5F).

### 3.11. Moringa Extract Reduces Expression of Lipogenic-Related Genes

Gene expression of *Srebf1*, *Fasn* and *Dagt2* were analyzed in liver since they are involved in lipid synthesis. *Srebf1*, *Fasn* and *Dagt2* mRNA levels are significantly higher in HF group than ND group (Figure 5G–I). Moringa administration significantly reduces *Srebf1* and *Fasn* (*p* < 0.01; Figure 5G,H) even with the HF diet administration. *Dagt2* mRNA levels in group treated with Moringa had a lower expression but did not reach statistical significance (Figure 5I).

### 3.12. Effect of Moringa Extract on The SIRT1/AMPKα/SREBP1C/FAS Signaling Axis and Protein

**Figure 6:**
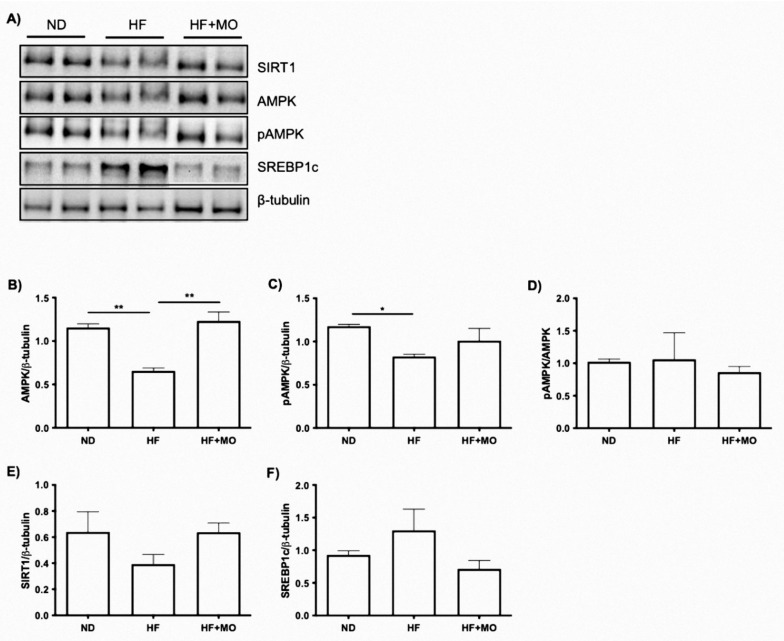
Effect of Moringa on the SIRT1/AMPKα/SREBP-1c/FAS signaling axis. (**A**) Representative image of the relative levels of protein of the SIRT1/AMPKα/p-AMPKα/SREBP-1c axis. (**B**) AMPKα (**C**) phosphorylated AMPKα, (**D**) Ratio p-AMPKα/AMPKα, (**E**) SIRT1, (**F**) and SREBP-1c. AMPK (*p* < 0.01) and pAMPK are increased in Mo group. Data are expressed as mean ± SE. * *p* < 0.05; ** *p* < 0.01.

We include WB analysis for SIRT1/AMPKα/SREBP1c/FAS since they are part of a signaling axis involved in fatty acid synthesis. Regarding SIRT1, a decrease in the HF group is observed compared to the ND group, and the HF + MO group had higher expression of SIRT compared to the HF group (Figure 6E). In addition, the AMPKα protein was found to be significantly increased in the ND group compared to the HF group, and the HF + MO treated group showed a significant increase compared to the HF group (*p* < 0.01; Figure 6B) similar with the ND group. Regarding the phosphorylated AMPKα protein, a tendency to increase is reflected between the group treated with Moringa compared to HF group (Figure 6C). However, the ratio pAMPKα/AMPKα between the groups did not show significant differences (Figure 6D). In the SREBP1c protein, no significant differences were found between the groups, but there is a tendency to decrease in the group treated with Moringa compared to the HF group (Figure 6F).

### 3.13. Effect of Moringa Extract on Liver Transcriptome in A MAFLD Model

**Figure 7:**
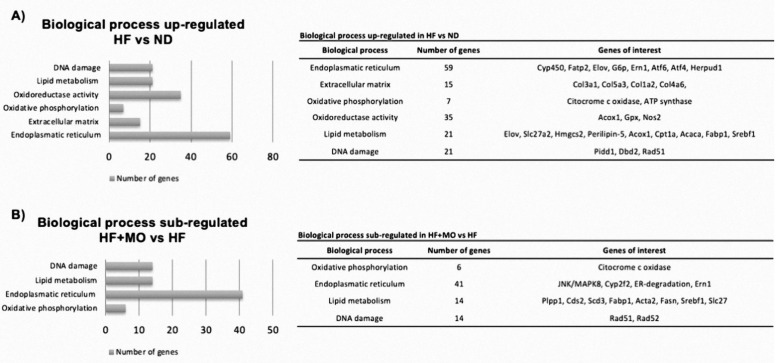

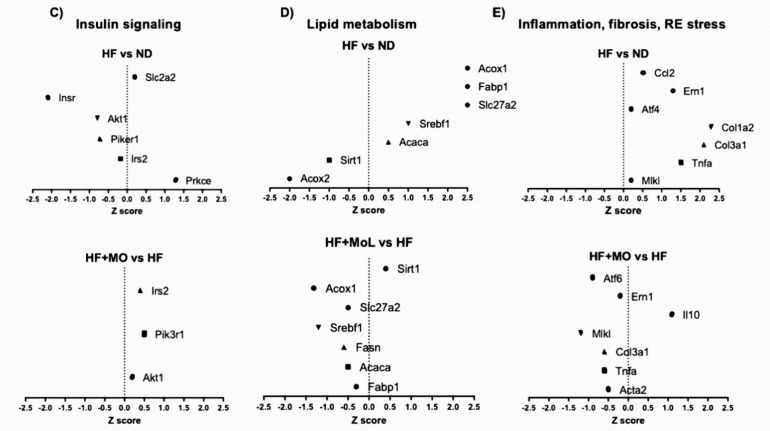
Transcriptome analysis of metabolic pathways modified after Moringa treatment. Pathways related with lipid metabolism, inflammation, oxidoreductase activity, oxidative phosphorylation, fibrosis, insulin signaling, RE stress, DNA damage and extracellular matrix are shown. (**A**) Metabolic pathways upregulated in HF samples are described and main genes listed. (**B**) Downregulation of specific genes in Mo treated animals are labelled. Graphs indicate genes detected in microarrays analysis regarding (**C**) Insulin signaling, (**D**) Lipid metabolism and (**E**) Inflammation, fibrosis and RE stress.

Based on data obtained from the transcriptome, over-regulated or under-regulated biological processes were found according to their gene ontology. Figure 7A shows the over-regulated biological processes of the HF group compared with the ND group, which are involved in MAFLD/NASH; the biological processes were: DNA damage (21 genes), lipid metabolism (21 genes), oxidoreductase activity (35 genes), oxidative phosphorylation (7 genes), extracellular matrix (15 genes) and endoplasmic reticulum stress (59 genes). The group treated with Moringa extract showed a down-regulation of genes involved in the biological processes of the endoplasmic reticulum stress (41 genes), lipid metabolism (14 genes), response to DNA damage (14 genes), and oxidative phosphorylation (6 genes) (Figure 7B). Alterations in genes involved in insulin signaling were observed in the HF group: negative regulation of the genes *Insr*, *Akt1*, *Piker1* and *Irs2*; positive regulation of *Slc2a2* and *Prkce*. Moringa treatment up regulated *Irs2*, *Piker1* and *Akt1* genes in contradiction of HF group (Figure 7C). In addition, lipid metabolism in the HF group was found to have negative regulation of the *Acox2*, *Sirt1* genes and positive regulation of *Fabp1*, *Slc27a2*, *Srebf1*, *Acaca* genes, compared to the ND group, the HF + MO group showed a negative regulation of the *Slc27a2*, *Srebf1*, *Fasn*, *Acaca*, *Fabp1* genes and a positive regulation of the *Acox2* and *Sirt1* genes against HF group (Figure 7D). Finally, the HF group showed positive regulation of genes involved in inflammation (*Ccl2, Tnfa*), fibrosis (*Col1a2* and *Col3a1*) and ER stress (*Ern1* and *Atf4*) compared to the ND group, the HF + MO group showed a negative regulation of *Tnfa* and positive regulation of *Il10*; involved in the inflammatory process; in addition, negative regulation of genes involved in fibrosis (*Col3a1* and *Acta2*) and ER stress (*Ern1* and *Atf6*) (Figure 7E) was also noted.

## 4. Discussion

This study deepens in the molecular mechanisms involved in the use of an aqueous extract of *Moringa oleifera* as a dietary supplement for the prevention of MAFLD/NASH progression. Daily administration of MO extract significantly abolishes animal weight gain and liver hyperplasia due to fat accumulation in animals fed with a high fat/carbohydrate diet, without any evident side effects. This result can be partially explained due to the reduction in food consumption and total kcal intake. This outcome was also reported by Bao et al. with the administration of niazirin, a phenolic glycoside isolated from MO seeds [10]. Besides, high fiber content in MO extract (around 11 g/100 g w/w) also decreased food ingest in this group [29]. However, some other mechanism could be also implicated in Moringa’s effects, such as the increase in thermogenesis reported by Waterman et al. [5]. We believe that de novo lipogenesis was diminished in animals treated with Moringa, since lipogenic genes were found downregulated including *Srebf1*, *Fasn and Dagt2*. These data match with reports from Bao et al. and Almatrafi et al. who used Moringa-derived niazirin compound and Moringa leaves [10,30]. Additionally, MO aqueous extract improve insulin sensitivity and HOMA-IR in our model. It has been reported that the expression of Srebf-1c in the liver is activated by insulin signaling [31], then Srebf-1c activation due to insulin signaling could be also affected in our Moringa treated animals via reduction of insulin levels.

Excessive fat feeding or fatty liver increases plasma levels of insulin, leptin, resistin and PAI-1 [32,33,34]. In our study, these serum markers were reduced in the groups treated with MO aqueous extract. These results agree with previous studies, where an aqueous extract of MO showed reduction in these parameters and antidiabetic effects in obese rats [5,8].

As known, consuming high-fat diet promotes production of pro-inflammatory cytokines such as IL1B and TNFA activated through NFkB pathway [35,36], as well as an increase in mitochondrial ROS production [32]. Free radicals cause lipid peroxidation on cell and organelle membranes and one of the measurable products of this reaction is malondialdehyde (MDA); a biomarker of oxidative stress [25]. Choi and Das demonstrated in a NAFLD model an increase in lipid peroxidation products such as MDA and 4HNE, due to the generation of reactive oxygen species [37,38]. In our study, the administration of Moringa reduces the production of MDA due to a decrease in ROS production. Then, antioxidant effect of our Moringa extract is confirmed. In the present study, main proinflammatory cytokines mRNAs levels were decreased—Tnfa, Il1b and Il6—with MO supplementation. This effect could be attributed to the antioxidant influence of MO flavonoids, such as quercetin [39], which inhibits the expression of pro-inflammatory cytokines by modulating NFkB transcriptional master gene [36].

Chronic liver inflammation leads to fibrosis progression, where inflammatory cells such as neutrophils and lymphocytes play a key role [40]. Likewise, growth factors (TGFβ, PDGF), cytokines (TNFα), adipokines (Leptin) and ROS are involved in the activation of HSC. These profibrogenic cells are responsible for the increased expression and secretion of collagen type I and III, and TGFβ1; and in consequence the overexpression of αSMA in cytoskeleton [35,41]. In our study, Tgfb1 and Col1a1 mRNAs decreased expression showed a potential antifibrotic effect of MO extract. This data correlated with Feustel et al. who reported a reduction in Tgfb1 mRNA level in Huh7 culture treated with MO, possibly due to the Moringa derived quercetin with ability to suppress HSC activation [42]. Hamza et al. also disclosed a reduced immunoreactivity in hepatic Col1a1 and Col1a3 in a rat model of CCl4-induced cirrhosis treated with a Moringa ethanolic extract [40].

We also analyzed SIRT1/pAMPKA/SREBP-1c signaling axis, involved in fatty acids synthesis, and we demonstrated that MO treatment increased SIRT1 protein expression and decreased SREBP-1c protein. SIRT1 is a deacetylase besides from being an energy sensor involved in aging and response to stress [43]. SIRT1 is required for maintenance of glucose and lipid homeostasis in the liver. In obesity and NAFLD models, SIRT1expression is decreased. Therefore, SIRT1 deacetylates and inhibits SREBP-1c activity, decreasing its stability and reducing its binding ability in the promoter region of lipogenic genes [44]. In several studies, the beneficial effects of SIRT1 activation against fatty liver disease have been verified [45]. Therefore, modulation of SIRT1 by our MO extract can also be a side mechanism that enriches beneficial effects observed in our study.

To the best of our knowledge, there is no previous evaluation of Mo effects in hepatic miRNAs expression. Since miR-122-5p is the most abundant miRNA in the liver [11] and modulates the metabolism of fatty acids, triglycerides, and cholesterol [46] we decide to analyze its expression, as well as, miR21a-50, miR103-3p and miR34a-5p, all involved in regulation of genes implicated in MAFLD development. After MO treatment, these miRNAs reached similar levels to normo-diet control animals and opposite to HF group. miRNAs expression is also affected by MO treatment and seems to be part of the molecular mechanism involved in its anti-lipogenic effects. Iliopoulos et al. and Gracia et al. showed that miR-122 overexpression induces an increased in SREBP1 protein, as it was found in our HF animals. Moreover, an increase was detected in miR-122 expression in a NAFLD model and specific genes involved in the progression of NASH through inhibition of the LKB1/AMPK pathway [19,47]. Furthermore, in a high-fat diet model, the treatment with a silibinin extract decreases miR-122 expression and improves liver healthiness [48]. These results are comparable with our data, where miR-122 decreased its expression after the treatment with Mo.

miR-21 is a known hepatic lipogenic and fibrotic miRNA [49]. The lack of *miR-21a-5p* decreases fibrogenesis through down-regulation of TGFβ signaling pathway [50]. Also, *miR-21a-5p* promotes lipid accumulation in the liver and progression to HCC through interaction in the *Hbp1-p53-Srebf1c* pathway on HF-fed mice showing an increase in *miR-21a-5p.* Its experimental knockdown prevented the accumulation of lipids in the liver [51]. These results harmonize with our data, where miR21 augment in HF model and decreased after MO treatment. This anti-lipogenic effect of miR21a-5p seems to be modulated by HBP1; a transcriptional activator of p53. p53 is an inhibitor of lipogenesis by constraining SREBP1c [51].

miR-103-3p regulates insulin sensitivity and glucose homeostasis and is highly expressed in the liver of patients with NAFLD [16]. Overexpression was also observed in our HF fed animals, and MO extract restored its expression to levels like ND group. This miR-103-3p overexpression in liver or fat results in Insulin Resistance (IR) by down-regulation of caveolin-1 [52]. Trajkovski et al. reported that silencing *miR-103-3p* in mice improves insulin sensitivity, effect also appreciated in our MO treated animals that decreased miR-103-3p expression and improve insulin sensitivity. Overexpression of *miR-103-3p* in hepatocytes also generates an increase in the expression of *Srebf1* [19]. Our results demonstrate that miR-103-3p and *Srebf1* were downregulated in MO group. Circulating miR-34a-5p levels are high in patients with MAFLD and in animal models of steatosis. Similarly, our data showed a higher hepatic miR-34a-5p expression in MAFLD model, while MO administration lessens its induction. In addition, a decrease in the expression of miR-34a-5p and an increase in its target genes *Sirt1* and *Ppara* have been reported when PFD was used against MAFLD development in a rodent model [53]; these results are in accordance with our data.

Key cellular processes are found to be altered in the development of non-alcoholic steatohepatitis (NASH). Using microarray analyses, we examined genes involved in lipid metabolism, inflammation, RE stress, fibrosis, insulin signaling, and how these metabolic pathways were modified in our MAFLD model after administration of MO extract. When comparing MO group to HF group, we found a decreased expression in genes involved in lipid metabolism, endoplasmic reticulum stress, DNA damage and oxidative phosphorylation, indicating MO display a beneficial modulation of these cellular functions. Specifically, we look for events involved in NASH development like insulin signaling and found a slight overexpression of the main genes involved in this via. Besides, many genes involved in lipid metabolism, RE stress and inflammation were also modulated in MO group. This transcriptome analysis, showed for the first time a wide spectrum of favorable effects of MO supplementation in this mouse model of NASH development that correlates with histological findings; since HF animals showed a significantly increased of inflammatory nodules and early fibrosis in liver tissue; while Moringa treated animals reduced inflammatory infiltrate, collagen staining and αSMA-positive cells. These findings lead us to endorse Moringa aqueous extract as a therapeutic approach to MALFD spectrum of liver disorders.

## 5. Conclusions

Moringa aqueous extract exhibited antioxidant, anti-lipogenic properties and modified gene expression in a model of MAFLD. Also, this therapeutic strategy improved insulin sensitivity and lipid metabolism, as well as epigenetic modifications implicated in MALFD. We believe Moringa extract can be of help in supplementing diet in humans to favor a spectrum of beneficial effects in metabolic-associated liver diseases.

## Figures and Tables

**Table 1:** Table of dietary intake during the experimental phases.

		ND	HF	HF + MO
Prior treatment				
Daily energy intake	Kcal	10.4 ± 1.42	13.5 ± 1.26 ^+++^	14.2 ± 1.26 ^++++^
Daily food intake	g	3.35 ± 0.47	2.49 ± 0.26 ^++++^	2.6 ± 0.25 ^+++^
Diet fat percentage	%	18	60	60
Daily fat intake	Kcal	1.80 ± 0.26	7.7 ± 0.8 ^++++^	8.03 ± 0.79 ^++++^
During treatment				
Daily energy intake	Kcal	10.2 ± 1.11	13.7 ± 1.18	11.5 ± 1.52 **
Daily food intake	g	3.28 ± 0.35	2.54 ± 0.21	2.1 ± 0.26 **
Diet fat percentage	%	18	60	60
Daily fat intake	Kcal	1.84 ± 0.20	7.7 ± 0.7	6.47 ± 0.79 *
Moringa Dose	mg/kg	-	-	0.29

The values represent the mean ± SE (*p* < 0.05). Comparison of the dietary consumption of the groups vs. the control group, +++ *p* < 0.001; ++++ *p* < 0.0001. Comparison of the HF + MO group before and during the treatment with the MO extract. * *p* < 0.05; ** *p* < 0.01.

## Data Availability

Not applicable.

## Supplementary Materials

The following are available online at https://www.mdpi.com/article/10.3390/nu14204225/s1, Table S1: TaqMan probes for miRNAs; Table S2. TaqMan probes specific for mRNAs; Table S3. Specific antibodies.
