# Supplementary material for: Moringa oleifera Improves MAFLD by Inducing Epigenetic Modifications
Source: Nutrients. 2022 Oct 11;14(20):4225. doi: 10.3390/nu14204225 (PMC9611907; doi:10.3390/nu14204225)
Supplement: Supplementary file 1 [file nutrients-14-04225-s001.zip › nutrients-1904688-supplementary.pdf]

Supplemental

Table S1. TaqMan probes for miRNAs

miRBase ID	Catalogue
mmu-miR-34a-5p	mmu481304_mir
mmu-miR-21a-5p	mmu482709_mir
mmu-miR-122-5p	mmu480899_mir
mmu-miR-103-3p	mmu478253_mir
mmu-miR-16-5p	mmu482960_mir

Table S2. TaqMan probes specific for mRNAs

Gen	Protein	Strain	Catalogue
Fasn	FAS	mice	Mm00662319_m1
Srebf1	SREBP	mice	Mm00550338_m1
Dgat2	DGAT2	mice	Mm00499536_m1
Tnfa	TNFA	mice	Mm00443258_m1
IL1b	IL1B	mice	Mm00434228_m1
IL6	IL6	mice	Mm00446190_m1
Col1a1	COL1A1	mice	Mm00801666_g1
Tgfb1	TGFB1	mice	Mm01178820_m1
Gapdh	GAPDH	mice	Mm99999915_g1

Table S3. Specific antibodies

Protein	KDA weigh	Origen	Catalogue
SIRT1	81	Mouse Monoclonal	ab110304
SREBP1c	122/70	Mouse Monoclonal	ad3259
AMPK	62	Mouse Monoclonal	ab80039
pAMPK	62	Rabbit mAb	2535
FAS	55	Mouse Monoclonal	sc-74540
CPT1A	88	Mouse Monoclonal	ab128568
NOS2	116	Mouse Monoclonal	sc-7271
4HNE	156	Rabbit polyclonal	ab46545
beta-tubulin	55	Polyclonal rabbit	gt-112141
